# Impact of reperfusion with blood venting on liver transplantation outcomes; a prospective case-control study 

**Published:** 2020

**Authors:** Nasir Fakhar, Abdolhamid Chavoshi Khamneh, Atabac Najafi, Ali Sharifi, Zeeshan Hyder, Javad Salimi

**Affiliations:** 1 *Liver Transplantation Research Centre, Imam Khomeini Hospital Complex, Tehran University of Medical Sciences, Tehran, Iran*; 2 *General Surgery Department, Hamadan University of Medical Sciences, Hamedan, Iran*

**Keywords:** Liver transplantation, Reperfusion, Mean arterial pressure

## Abstract

**Aim:**

This study aimed to evaluate the impact of two different reperfusion techniques on outcomes of LT patients.

**Background:**

Post-reperfusion syndrome (PRS) during liver transplantation (LT) remains a serious issue for both the surgeon and anesthetist.

**Methods:**

In this prospective study, all liver transplant recipients referred to the liver transplantation department of Imam Khomeini Hospital, Tehran, Iran, from January 2016 to June 2017 were enrolled in the study and were divided into two groups of vented (reperfusion with 300cc blood venting) and non-vented (reperfusion without blood venting) cases. Then, 30-minute intraoperative hemodynamic and biochemical changes, as well as 2-month complications and 6-month mortality, were compared between the groups.

**Results:**

57 LT cases (31 vented and 26 non-vented) were studied (50.9% female). The two groups had a similar age (p = 0.107), sex (p = 0.885), MELD score (p = 0.61), donor warm ischemic time (p = 0.85), recipient warm ischemic time (p = 0.36), cold ischemic time (p = 0.99), comorbid disease (p = 0.502), and etiology of end-stage liver disease (p = 0.281). PRS occurred in 3 (11.5%) patients in the vented group and 4 (12.9%) in the non-vented group (p = 0.69). One (3.8%) patient in the non-vented group and 4 (12.9%) patients in vented group died (p = 0.229).

**Conclusion:**

Reperfusion with and without blood venting had the same outcome regarding intraoperative hemodynamic and biochemical changes, PRS rate, and postoperative complications, as well as 6-month survival. Thus, it seems that blood venting is not a necessary method for decreasing post-reperfusion complications following LT.

## Introduction

 Liver transplantation (LT) was first attempted in 1963, and the first successful LT was performed in 1967 by Dr. Starzl at the University of Pittsburgh (1). Achievements in surgical and preservation techniques and progress in immunosuppression have significantly improved survival of patients and made LT the gold standard of treatment for patients with end-stage liver disease worldwide (2, 3).

Despite these progressions, some issues such as hemodynamic disturbances during LT, especially after reperfusion, remain a serious concern for surgeons and anesthetists (4).

Post-reperfusion syndrome (PRS) was described as cardiovascular collapse after reperfusion of the graft by Aggarwal et al. They defined a syndrome of severe cardiovascular dysfunction, bradycardia, decreased mean arterial pressure (MAP), and systemic vascular resistance with a simultaneous increase in pulmonary filling pressures (5). 

Numerous changes in electrolytes and inflammatory mediators such as various cytokines after reperfusion have been studied in this regard (6, 7). In a review by Siniscalchi et al. possible risk factors for PRS were divided into three categories of donor/organ-related, recipient related, and procedure-related (8).

It has been hypothesized that backward (retrograde) reperfusion from vena cava causes more gradual rewarming of the graft and allows the grafted liver to be perfused initially by low-pressure and low oxygenated blood, causing diminished production of free oxygen radicals. These effects along with the reduction in blood loss, by eliminating vena cava blood venting, improve hemodynamic stability and reduce the incidence of PRS, as observed by some authors        (9, 10).

In our center, we routinely use two different surgical methods for reperfusion based on surgeon preferences: ante-grade reperfusion via the portal vein with 300 mL blood venting and retrograde reperfusion through the vena cava without blood venting. This study aimed to evaluate the impact of the two different reperfusion techniques on outcomes of LT patients. 

## Methods

***Study design and setting***

In this prospective study, candidate patients for LT referred to the liver transplantation department of Imam Khomeini Hospital, Tehran, Iran, from January 2016 to June 2017 were enrolled in the study. They underwent transplantation using vented or non-vented reperfusion methods based on the in-charge surgeon’s preferences. Then, intra-operative hemodynamic and biochemical changes, as well as early postoperative graft function and complications, were compared between the groups. The study protocol was approved by the Ethics Committee of Tehran University of Medical Sciences (number IR.TUMS.IKHC.REC.1396.2441). Researchers adhered to the Helsinki’s declarations regarding the confidentiality of patients’ profiles. Also, informed written consent was obtained from patients or their legal guardians.

***Participants***

Patients with end-stage liver disease who were candidates for LT were enrolled in the study without any age or sex limitation. Re-transplantation, combined liver/kidney transplantation, and living-donor related transplantation cases were excluded from the study.

***Data gathering***

Using a predesigned checklist, demographic data, indication for liver transplantation, comorbidities, Model for End-Stage Liver Disease (MELD) score, donor warm ischemic time (time between donor aorta cross-clamp and liver placement in ice bag), graft cold ischemic time, and recipient warm ischemic time (time between graft withdrawal from ice and reperfusion), and intraoperative data (amount of packed cells and platelets transfused as well as fibrinogen infused, hemodynamic and biochemical changes, etc.), as well as outcomes (PRS, survival, and post-operative complications) were recorded for all patients. All data were recorded by a liver transplantation fellow, who was present in the operation room during all the performed transplants. 

***Definitions***

Primary non-function (PNF), hepatic artery and portal vein thrombosis, rejection (biopsy-proven), need for dialysis, biliary problems, infection (pneumonia and wound infection) were considered as postoperative complications.

Hemodynamic parameters (mean arterial pressure, heart rate, cardiac output, and systemic vascular resistance) and biochemical variables (serum potassium level and arterial blood gas parameters including pH and HCO_3_) were recorded before de-clamping and on the 1^st^, 5^th^, 15^th^ and 30^th^ minutes post-reperfusion. 

PRS was defined as more than 30% drop in the mean arterial pressure and/or heart rate on the 1^st^ and/or 5^th^ minutes after reperfusion in comparison with pre-de-clamping    (11).

***Surgical technique***

LTs were performed routinely by Piggyback or Standard hepatectomy technique. During implantation, the graft was flushed with 1 liter of room temperature Ringer solution in both groups. After caval and portal anastomosis completion, in the vented group, ante-grade graft reperfusion through the portal vein was established and 300 mL blood was vented out from the donor’s bottom cava before caval de-clamping. Indeed, the inferior cavocaval anastomosis was left loose using a right-angle instrument kept between the sutures and the suction tip close to it. Then, the de-clamping of the portal vein was performed and blood was suctioned around the anastomosis site and stopped when the suction bottle contained 300 mL blood. After 300 mL suction of blood, the anastomosis sutures were tightened. In the non-vented group, retrograde reperfusion was established through the supra-hepatic vena cava without portal vein blood venting. The rest of the operative techniques were the same in both groups.

***Outcomes***

Hemodynamic changes after reperfusion were considered as primary outcomes while other evaluated outcomes such as biochemical changes after reperfusion, postoperative complications (during the 2-month follow up), and 6-month survival of the patients were regarded as secondary outcomes.

***Statistical Analysis***

Statistical analysis was performed using IBM SPSS 23. For continuous variables mean ± standard deviation (SD) and for categorical data frequency (percentage) were reported. To compare categorical variables based on the venting status (yes or no), the chi-square test was used. To compare mean values of continuous variables, independent t-test was applied for normally distributed variables and Mann-Whitney U test for abnormal data. Then, to evaluate the effects of time and intervention as well as their combination, repeated measured ANOVA was employed. To evaluate the effects of time, post-hoc pairwise comparisons were used and Bonferroni adjustments were reported. P-value < 0.05 was considered as level of significance. 

## Results

***Baseline Characteristics of Studied Patients***

Fifty-seven cases of liver transplantation (31 cases of vented and 26 non-vented) with the mean age of 40.67 ± 16.08 (8-69) years were studied (50.9% female). Table 1 summarizes and compares the baseline characteristics of the studied patients. The two groups had similar conditions regarding age (p = 0.107), sex (p = 0.885), MELD score (p = 0.61), donor warm ischemic time (p = 0.85), recipient warm ischemic time (p = 0.36), cold ischemic time (p = 0.99), comorbid disease (p = 0.502) and etiology of end-stage liver disease (p = 0.281). The mean age (31.14 ± 13.86 vs 32.80 ± 19.11 years; p = 0.810) and sex (p = 0.558) of the donors were the same in both groups.

***Outcomes***

***Hemodynamic changes***

**Figure 1:**
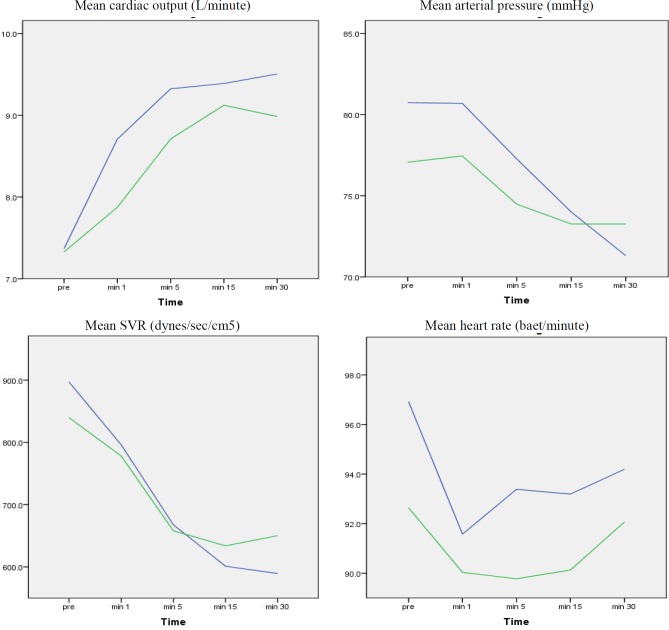
Mean hemodynamic parameter changes before and 1, 5, 15, and 30 minutes post-reperfusion; Blue line = non-vented and green line = vented; SVR: systemic vascular resistance. Differences were not significant in the four comparisons (p > 0.05)

Figure 1 compares the changes in hemodynamic parameters of patients before and on the 1^st^, 5^th^, 15^th^ and 30^th^ minutes post-reperfusion. There was no significant difference between the groups regarding the mean arterial pressure (p > 0.20), mean heart rate (p> 0.39), mean systemic vascular resistance (p > 0.26) and mean cardiac output (p > 0.20) at any measured time. PRS occurred in 3 (11.5%) patients in vented and 4 (12.9%) patients in non-vented group (p = 0.69). One of the 7 (14.2%) PRS cases died in the vented group (p = 0.494). 

***Laboratory parameter changes***

There were no significant differences between groups regarding arterial blood gas parameters such as pH (p > 0.52) and Hco3 (p > 0.11) before and 1, 5, 15^,^ and 30 minutes post-reperfusion. The amounts of packed cells (p = 0.774), platelets (p = 0.374) and fibrinogen (p = 0.998) requirements during operation were the same between the two groups. There was no significant difference either in pre-operative (p = 0.516) and post-operative hemoglobin levels on the 1^st^(p = 0.700) and 3^rd^(p = 0.680) days after the operation between the vented and non-vented groups.

The mean potassium level was significantly lower in the non-vented group before (3.8 vs 4.3; p = 0.025) as well as at 1 (4.1 vs 4.7; p = 0.023), 5 (3.4 vs 3.8; p = 0.026) and 15 (3.4 vs 3.8; p = 0.0.20) minutes post-reperfusion. This difference was statistically non-significant 30 (3.6 vs 3.9; p = 0.21) minutes following reperfusion. On the other hand, the pattern of serum potassium changes was the same in both groups. 

***Post-operative complications (2-month follow up)***

The frequency of primary non-function (p = 1.00), hepatic artery thrombosis (p = 1.00), portal vein thrombosis (p = 0.44), biopsy proven rejection (p = 1.00), need for dialysis (p = 1.00), biliary complications (p = 1.00), wound infection (p = 0.08) and pneumonia (p = 1.00) was the same in the two groups (table 2). 

**Table 1:** Baseline characteristics of studied patients

Variables	Vented (n=31)	Non-vented (n=26)	P-value
Age (years)	43.90 ± 13.53	36.81 ± 18.20	0.107
Sex			0.885
Male	16 (51.6)	12(46.2)	
Female	15 (48.4)	14 (53.8)	
MELD score (mean± SD)	22.00 ± 5.854	23.29 ± 7.653	0.61
Times (minutes)			
Donor warm ischemic	22.16 ± 8.47	23.08 ± 11.46	0.85
Recipient warm ischemic	39.35 ± 9.21	36.48 ± 7.52	0.36
Cold ischemic	284.16 ± 50.44	287.62 ± 55.47	0.99
Etiology of ESLD			0.281
Autoimmune hepatitis	3 (9.7)	6 (23.1)	
Cryptogenic	6 (19.4)	3 (11.5)	
Hepatitis C virus (HCV)	3 (9.7)	0 (0.0)	
Hepatitis B virus (HBV)	3 (9.7 )	2 (7.7)	
Wilson's Disease	3 (9.7)	1 (3.8)	
Primary biliary cirrhosis	1 (3.2)	1 (3.8)	
Primary sclerosing cholangitis (PSC)	4 (12.9)	0 (0.0)	
Acute liver failure	3 (9.7)	4 (15.4)	
Non-alcoholic steatohepatitis	2 (6.5)	0 (0.0)	
Budd chiari syndrome	1 (3.2)	1 (3.8)	
Hepatocellular carcinoma (HCC)	0 (0.0)	1 (3.8)	
HCV+ HCC	0 (0.0)	1 (3.8)	
Autoimmune hepatitis +HCC	0 (0.0)	1 (3.8)	
Cryptogenic + HCC	0 (0.0)	1 (3.8)	
Hepatocellular carcinoma (HCC)	0 (0.0)	1 (3.8)	
Budd chiari syndrome + PSC	0 (0.0 )	1 (3.8 )	
Acute on chronic hepatic failure	1 (3.2)	0 (0.0 )	
Others	1 (3.2)	3 (11.5)	

Data are presented as mean ± standard deviation or frequency (%); ESLD: end-stage liver disease, MELD: Model for End-Stage Liver Disease

**Table 2:** Comparison of post-operative complications between the two groups

Post-operative complications	Vented	Non-vented	P
Primary non-function	2 (6.5)	2 (7.7)	1.00
Hepatic artery thrombosis	0 (0.0)	0 (0.0	1.00
Portal vein thrombosis	0 (0.0)	1 (4.0)	0.44
Rejection (biopsy proven)	3 (9.7)	2 (8.0)	1.00
Need for dialysis	3 (10.0)	2 (8.3)	1.00
Biliary complications	1 (3.2)	1 (3.8)	1.00
Wound infection	0 (0.0)	3 (13.0)	0.08
Pneumonia	5 (16.7)	4 (16.0)	1.00

Data are presented as mean ± standard deviation or frequency (%)

***Six-month survival***

One (3.8%) patient in the non-vented group and 4 (12.9%) patients in the vented group died (p = 0.229). The cause of death was the primary non-function for 3 (60%) cases and sepsis as well as severe dysfunction for 2 (40%) cases. The mean duration of hospital stay was 14.00 ± 7.60 days in the vented and 11.23 ± 7.34 days in the non-vented group (p = 0.04). In addition, the mean operation time was 259.69 ± 37.17 minutes in the non-vented and 271.00 ± 42.05 minutes in the vented group (p = 0.291).

## Discussion

Based on the findings of the present study, liver transplantation patients undergoing reperfusion with and without 300cc blood venting had similar outcomes regarding 30-minute hemodynamic and biochemical changes, PRS development, 2-month post-operation complications, as well as 6-month survival. Lower serum potassium level during the first 15-minute post-reperfusion in the non-vented cases was the only significant difference between the groups in this study.

Many surgical interventions have been attempted to minimize the severity of cardiovascular disturbances following reperfusion of the liver (9, 12, 13). In a study by Brems et al., less hemodynamic instability was reported in the group with systemic reperfusion without vena cava venting (14). Millis et al. found lower incidence of PRS in patients undergoing portal vein flush without venting (15).

Portal vein flush without vena caval blood venting caused a lower incidence of hemodynamic changes in the peri-operative period following liver transplantation and earlier recovery of the graft function in a study by Gruttadauria et al. (12).

More recently, in a study on 478 patients by Fukazawa et al., crystalloid flush with backward reperfusion and portal blood flush with forward reperfusion were compared. They found a significant decrease in the primary non‐function, cardiac arrest and PRS, along with an increase in 30‐day graft survival in crystalloid flush with backward reperfusion    (10).

Performing a comprehensive reviewof graft flush prior to reperfusion in liver transplantation, Houben P. et al. found that flushing protocols were usually based on personal or institutional experience. They concluded that the available literature does not provide a final appraisal of the benefits of graft flush in liver transplantation (16).

Another review study by Gurusamy KS et al. in 2012 compared different methods of flushing and reperfusion during liver transplantation. It found no significant difference in mortality, graft survival, or severe morbidity rates between the groups  (17).

In an unpublished retrospective study, we did not find any significant benefit for blood venting. In this study, factors that were proposed to have a role in PRS and short-term transplant outcomes, including donor and recipient warm ischemic time, graft cold ischemic time, donor and recipient age, operation time, intraoperative blood products transfusion, and MELD score were similar between the groups. 

We noted a steady decline in systemic vascular resistance over 30 minutes after de-clamping along with an increase in the cardiac output in both groups. Also, we found a notable reduction in the heart rate on the first minute after de-clamping in both groups. Heart rate decreased until 5 minutes after de-clamping in the vented group but then became stable or increased over time. There was no continued fall in the heart rate in the non-vented group after the first minute.

We noted a moderate rise in the serum potassium on the first minute followed by a marked declinein the fifth minute after de-clamping in both groups. On the other hand, the pattern of these changes was the same between vented and non-vented groups. This was in contrast with the Millis et al. study, which reported a smaller percentage of increase in serum potassium after de-clamping in the venting method (15).

Considering PRS being defined as more than 30% drop in the mean arterial pressure or heart rate in the first five minutes after de-clamping, we did not find any significant difference between the two groups in this regard.

PRS incidence varies largely among the considered studies, ranging from 12% to 77% (8). In our experience, it was 11.5% and 12.9% in vented and non-vented groups, respectively. It seems that the most important cause of this wide range is the large difference between PRS definitions. Similarly, other factors such as patient population and surgical techniques varied considerably across studies. This heterogeneity could influence the rate of PRS found, which explains the vast difference between PRS incidences presented in different studies. The low rate of PRS in our study may be due to shorter ischemic times and better situation of donors. 

Since the outcomes of vented and non-vented reperfusion methods in liver transplantation have been similar, it seems that blood venting could be omitted as a method for reducing post de-clamping complications such as PRS.

Short duration of follow-up, small sample size, researchers being aware of the type of procedure, and non-random allocation of patients based on the in-charge surgeon preferences were among the most important shortcomings of the present study.

Reperfusion with and without blood venting had similar outcomes regarding 30-minute hemodynamic and biochemical changes, rate of PRS development, 2-month post-operation complications, and 6-month patient survival. Thus, it seems that vena caval blood venting could be omitted as a method to decrease post-reperfusion complications following liver transplantation.
